# Emergence in Elderly Patient Undergoing General Anesthesia with Xenon

**DOI:** 10.1155/2013/736790

**Published:** 2013-05-22

**Authors:** Maria Sanfilippo, Ahmed Abdelgawwad Wefki Abdelgawwad Shousha, Antonella Paparazzo

**Affiliations:** Department of Anesthesiology and Intensive Care, Sapienza University, Viale del policlinico 155, 00161 Rome, Italy

## Abstract

*Introduction*. It is a consensus that the postoperative cognitive function is impaired in elderly patients after general anaesthesia, and such category patient takes more time to recover. Xenon is a noble gas with anesthetic properties mediated by antagonism of N-methyl-D-aspartate receptors. With a minimum alveolar concentration of 0.63, xenon is intended for maintaining hypnosis with 30% oxygen. The fast recovery after xenon anaesthesia was hypothesized to be advantageous in this scenario. *Case Presentation*. We report the case of 99-year-old woman who underwent sigmoid colon carcinoma resection with colorectal anastomosis. We carried out the induction phase by propofol, oxygen, fentanil, and rocuronium bromide, and then we proceeded to a rapid sequence endotracheal intubation consequently. The patient was monitored by IBP, NIBP, ECG, cardiac frequency, respiratory rate, capnometry, TOF Guard, blood gas analysis, and BIS. For maintenance we administrated oxygen, remifentanil, rocuronium bromide, and xenon gas 60–65%. Shortly after the end of surgery the patients started an autonomous respiratory activity, and a high BIS level was also recorded. Decision was made by our team to proceed into the emergence phase. The residual neuromuscular block was antagonized by sugammadex, modified Aldrete score was implicated, and we got our patient fully awake without any cognitive dysfunction or delirium. *Conclusion*. The rapid emergence to full orientation in very elderly patient who had been anesthetized by xenon shows concordance to the high BIS values and the clinical signs of the depth of anesthesia.

## 1. Introduction

Aging is an irreversible and progressive physiological phenomena characterized by degenerative changes in the structure and functional reserve of organs and tissues [1]. Advances and improvement in medical science have increased life expectancy, and thus perioperative surgery and anesthesia in the elderly patient have become an extremely important issue.

Elderly patients (arbitrarily defined as being over 65 years of age) are vulnerable to the adverse effects of anesthesia because of their reduced margin of safety. Morbidity and mortality increase with advancing age, with a steep increase after the age of 75 years [2]. The frequency of complications related to anesthesia is 0.5% in patients >80 years old [3].

Elderly patients often take more time to recover completely from the central nervous system effects of general anesthesia, particularly if they were confused or disoriented preoperatively.

Over the last decade there has been renewed interest in the use of xenon as an anaesthetic, and xenon has been licensed as an anesthetic in Europe since 2005. Xenon potently inhibits N-methyl-D-aspartate (NMDA) noncompetitively, with little effect on GABAA receptors or non-NMDA glutamatergic receptors.

The main beneficial features of xenon anesthesia are fast induction and emergence because of low solubility in blood and tissues, along with remarkably stable hemodynamics even in patients with impaired cardiac function.

Xenon has proven to be a safe and well-tolerated anesthetic in clinical trials [4, 5].

In comparison with nitrous oxide, Goto and colleagues found that emergence from xenon anesthesia is 2 or 3 times faster than that from comparable MACs of nitrous oxide/isoflurane and nitrous oxide/sevoflurane anesthesia. Furthermore, xenon compares favorably with other anesthetic agents [6].

## 2. Case Presentation

A 99-year-old female (height 1.60 m; weight 45 kg, ASA III) was admitted to our hospital and underwent major abdominal surgery (sigmoid colon carcinoma resection with colorectal anastomosis) under general anesthesia, and the surgery duration was about 220 minutes. Her medical history revealed mild degree heart failure, chronic normocytic anemia, gastritis, and allergy for NSAIDs and penicillin antibiotics.

Her preoperative laboratory tests: hemoglobin 8.8 mg·dL^−1^, hematocrit 30.9%, leukocytes 11,500 mm^−3^ without deviation, platelets 453,000 mm^−3^, sodium 131 mg·dL^−1^, potassium 4 mg·dL^−1^, magnesium 0.58 mg·dL^−1^, creatinine 0.5 mg·dL^−1^, and total calcium 8.24 mg·dL^−1^.

In the preoperative room we prepared our patient by antibiotics prophylaxis: ciprofloxacin 2 gm; metronidazole 500 mg and an antiemetic agent; ondansetron 4 mg.

Our patient was monitored by pulse oximetry, expiratory capnography, invasive and noninvasive blood pressure, electrocardiogram, bispectral index (BIS), neuromuscular transmission (TOF Guard), and diuresis.

A peripheral venous access (20 G) was established in upper right limb, and also a central venous access was done immediately after the induction phase as it was needed for postoperative chemotherapy afterwards.

We induced our anesthesia by oxygen, propofol 50 mg, fentanil 100 mcg, and rocuronium bromide 30 mg, and then we proceeded to a rapid sequence endotracheal intubation (tube diameter was 6.5 mm),

The maintenance of the anesthesia was achieved by continuous infusion of remifentanil in a dose of 0.2 mcg/kg/min, rocuronium bromide 10 mg, xenon 60–65%, and O_2_ 35–40%, the fluid replacement was calculated depending on her diuresis, plasma fluid, intraoperative blood loss, and anemia, and she was refunded by ringer lactate 1500 mL, Nacl 0.9% 1000 mL, fresh plasma fluids 1000 mL, and packed red cells 500 mL.

A closed-circuit anesthesia machine (Felix Dual, Taema) was used for xenon gas delivery. The ventilation parameters were the following: “pressure-cycled mechanical ventilation with inhalation pressure indexes of 19 cm H_2_O, respiratory frequency 12 incursions per minute, PEEP 5 cm H_2_O, FiO_2_ 35–40%, and inspiratory/expiratory time ratio 1 : 2, and the exhaled tidal volume was around 380 mL.”

Blood gas analysis was performed twice: 30 minutes after the start and 30 minutes before the end of surgery.

 We registered electrolyte disorders, and they were resolved by administration of KCL 40 mEq and Ca^++^ gluconate in dose of 1 g·10 m^−1^ (Tables 1 and 2).

After 5 minutes from the end of the surgery we noticed our patient starting a voluntary respiratory activity with a high BIS level, so we decided to proceed for the emergence phase and extubation reversing the neuromuscular blocking by sugammadex in a dose of 100 mg [7] with careful monitoring for her cardiovascular and respiratory functions. Both modified Aldrete score and BIS values were recorded (Table 3).

After 13 minutes we got the complete recovery of our patient, she was awake without any confusion state, delirium, or cognitive dysfunction, also she had excellent and stabile both hemodynamic and respiratory functions, postoperative pain control was achieved by continuous intravenous infusion of morphine (5 mg over 24 h), and she was transferred to ICU for close monitoring. 

## 3. Conclusion

In our case we got surprised for the rapid emergence of our patient; this corresponds with the low blood gas partition coefficient of xenon (0.115). We also concluded that the BIS values show sufficient concordance with clinical signs of anesthetic depth and the emergence to full orientation in very elderly patient who had been anesthetized by xenon, and consequently we encourage more studies concerning the use of xenon in general surgery with such category of patients [8–11]. 

## Figures and Tables

**Table 1:** Hemogasanalysis 30 minutes after the start of the surgery.

Status:	Accepted	
Analysis:	25/01/2013 09:32:29	
Sample Type:	Arterial	

S/N:	08071675	

Measured (37.0°C)		
pH	7.51	
*p*CO_2_	31	mmHg
*p*O_2_	187	mmHg
Na^+^	134	mmol/L
K^+^	*3.1 *	mmol/L
Ca^++^	1.08	mmol/L
Glu	125	mg/dL
Lac	0.9	mmol/L
Oximeter		
tHb	9.0	g/dL
O_2_Hb	96.2	%
COHb	2.1	%
MetHb	1.8	%
HHb	−0.1	%
sO_2_	100.1	%
Derivatives		
TCO_2_	25.7	mmol/L
BEecf	1.7	mmol/L
BE (B)	1.9	mmol/L
Ca^++^ (7.4)	1.13	mmol/L
sO_2_ (c)	99.7	%
HCO_3_^−^ (c)	24.7	mmol/L
HCO_3_^−^ (std) _standard_	26.4	mmol/L
Hct (c)	27	%
Inserted		
Temp	37.0	°C
O_2_/Vent		
FIO_2_	35.0	%

**Table 2:** Hemogasanalysis 30 minutes after the end of the surgery.

Status:	Accepted	
Analysis:	25/01/2013 13:03:35	
Sample Type:	Arterial	

S/N:	08071675	

Measured (37.0°C)		
pH	7.33	
*p*CO_2_	45	mmHg
*p*O_2_	97	mmHg
Na^+^	131	mmol/L
K^+^	5.0	mmol/L
Ca^++^	1.25	mmol/L
Glu	178	mg/dL
Lac	2.3	mmol/L
Oximeter		
tHb	9.7	g/dL
O_2_Hb	95.0	%
COHb	2.2	%
MetHb	1.8	%
HHb	1.0	%
sO_2_	99.0	%
Derivatives		
TCO_2_	25.1	mmol/L
BEecf	−2.2	mmol/L
BE (B)	−2.3	mmol/L
Ca^++^ (7.4)	1.21	mmol/L
sO_2_ (c)	97.0	%
HCO_3_^−^ (c)	23.7	mmol/L
HCO_3_^−^ (std) _standard_	23.1	mmol/L
Hct (c)	29	%
Inserted		
Temp	37.0	°C

**Table 3:** BIS: bispectral index.

Time	1 min	5 min	10 min	13 min
BIS	35	92	97	99
Modified Aldrete	5	7	9	10
